# Effects of Test Location and Sample Number on the Liking Ratings of Almond Beverage and Vegan Ramen Products

**DOI:** 10.3390/foods12030632

**Published:** 2023-02-02

**Authors:** Jae-Yeon Yoon, Han-Sub Kwak, Mi-Ran Kim, Seo-Jin Chung

**Affiliations:** 1 Department of Nutritional Science and Food Management, Ewha Womans University, Seoul 03760, Republic of Korea; 2 Food Processing Research Group, Korea Food Research Institute, Wanju-gun 55465, Republic of Korea; 3 KFRI School, University of Science and Technology, Wanju-gun 55465, Republic of Korea; 4 Department of Food Science and Nutrition, The Catholic University of Korea, Bucheon-si 14662, Republic of Korea

**Keywords:** laboratory condition test, home-used test, test location, sample number, almond beverage, vegan ramen

## Abstract

The present study investigated the effects of the evaluation environment and sample number on liking ratings within the same testing session. It comprised two experiments that determined consumer taste ratings of the following food products: (1) almond beverage and (2) vegan ramen, as rated by 322 and 287 Korean consumers, respectively. Consumers tasted each food product under either laboratory or home-used test conditions. Additionally, three levels of sample numbers were established for evaluation (almond beverage test: 1, 2, and 4; vegan ramen test: 1, 3, and 5) in each test condition. A target sample was selected for each of the two food products to directly ascertain the effects of the evaluation environment and sample number on the liking ratings. The results revealed that during the same evaluation session, the sample number affected the liking ratings of the target sample more than the testing location. Moreover, the sample number effect was product item dependent, that is, no significant change was noted in the liking ratings of the target almond beverage sample according to sample number, whereas significant differences were observed in the liking ratings of the target vegan ramen sample. Furthermore, the sample number effect was more prominent under laboratory test conditions than under home-used test conditions probably due to the serving order effect driven by hedonic contrast, carry over effect, and sensory specific satiety. The findings demonstrate that home-used tests should be recommended over laboratory tests when measuring the liking of a small number of multiple sample food items with high flavor complexity.

## 1. Introduction

Consumer taste tests are often conducted either under laboratory conditions (laboratory condition test, LT) or in the home environment (home-used test, HUT). The LT is advantageous in that it allows the experimenter to control the sample preparation process, evaluation procedure, and other factors that may affect sensory evaluation. Thus, factors not of interest to the investigation, such as noise, can be minimized or eliminated. It can also be considered a cost- and time-effective method, as taste testing can be completed within a brief period. However, since a laboratory environment is not where consumers normally consume food, the representativeness of the data remains questionable [1]. Additionally, since the amounts of consumed and exposure time of sample are limited in LT, the results can be erroneous [2]. In contrast, the HUT potentially yields more realistic data because it entails data collected from a more “real-life” environment. Notwithstanding, the method’s drawbacks include practical impediments and biases, such as sample repackaging, sample shelf life, and uncertainty in the evaluation process owing to an uncontrolled environment, among others. To circumvent these practical challenges, the LT tends to be used more frequently than the HUT.

From 2020 to 2022, the COVID-19 pandemic normalized social distancing, preventing people from gathering in one place; hence, most social interactions were conducted in a non-face-to-face manner. This critically affected experimental design in sensory science, as taste testing under laboratory conditions could not be readily conducted. Hence, the HUT became the preferred option over the LT. Additionally, the drive-through and video-guided sensory tests, among others, have recently emerged as alternatives to LTs [3,4,5,6].

Studies have shown that physical environments influence consumer hedonic scores of food products [7,8]. Studies have also compared food liking scores collected under controlled (e.g., LT and central location test (CLT)) and natural (e.g., HUT) conditions [1,9,10,11]. While certain studies did not observe significant environmental effects on food hedonic ratings [1,12,13,14,15], the majority reported significant evaluation environmental effects. In many cases, HUT scores have generally exceeded LT scores [9,10,11,16,17,18,19]; nonetheless, conflicting results have also been obtained [20,21]. A couple of studies found that significant environmental effects were product type dependent [22,23].

One of the differences between LT and HUT conditions is the sample number typically tested during an experiment. In laboratory testing, the sample number evaluated during a session is not strictly limited, provided the samples do not fatigue subjects. It is usually recommended that 5–6 samples be evaluated in one session of LT [24], and a previous study reported that up to 12 samples can be evaluated in one session by consumers with no adverse effect [25]. However, the testing of a small number of samples is generally recommended for the HUT, usually 1–2 samples [24,26], since a large sample number potentially confuses subjects in terms of sample preparation and following evaluation instructions. Studies have demonstrated that the sample number evaluated in one session affects the discrimination of samples and hedonic ratings. Whereas subjects become less sensitive due to adaptation and fatigue when multiple samples are evaluated together, and they can become more sensitive with the judges’ experience and increased frame of reference [27]. When multiple samples are evaluated together within a session, the contrast effect, wherein a product with a low acceptance/intensity level is assigned a lower rating when evaluated after one with a high acceptance/intensity level, easily manifests [28] and potentially induces sequential bias [29]. Usually, the LT uses a sequential, monadic design within a session (single-session, multi-sample design); however, the HUT provides an adequate time period, usually 4–7 days for one sample [26], for repeated same-sample or different-sample evaluation over a few days (multi-session, single-sample design) [9]. 

The present study aimed to investigate the effects of the evaluation environment and sample number in a test set on food product liking ratings. To investigate these two factors, food items were selected based on their degree of familiarity and sensory complexities. The hedonic ratings of foods with high familiarity have been found to exhibit a considerable degree of consistency, regardless of the evaluation environment [1] and other contextual factors [30], while those of unfamiliar foods have proven more susceptible to contextual factors [31]. Flavor and the perceived complexity of flavor potentially influence sensory–perception changes [32]. A number of studies have analyzed the link between complexity and hedonic response [33,34]. We selected vegan products, namely almond beverage and vegan ramen, as the food items of interest. The vegan food sector is relatively new; nonetheless, its market size is growing rapidly in Korea and other countries, as consumer demand for sustainable foods that considers animal welfare, health, and environment, among others, continues to rise [35]. Almond beverage has relatively low complexity because it is a liquid food with a mildly sweet, nutty flavor, whereas ramen can be considered high in complexity since it consists of soup and noodles carrying umami, salty, and spicy flavor properties.

## 2. Materials and Methods

### 2.1. Experimental Design

**Figure 1:**
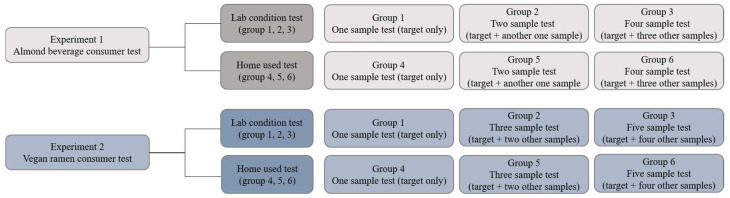
Overall experimental design.

This study comprised two sub-experiments: (1) the almond beverage and (2) vegan ramen consumer tests. As shown in Figure 1, each experiment involved six experimental groups. The groups were factorial arrangements of two evaluation environments (LT and HUT) and three evaluation sample numbers (almond beverage test: one, two, and four; ramen test: one, three, and five). The sample numbers for investigation were not equivalent between the almond beverage and ramen experiments, since the brands available for each food item in Korea differ. 

A target sample was selected to investigate the effect of sample number on the liking rating. The target sample was included in all six experimental groups, and liking scores were compared between the two evaluation environments and among the various evaluation sample numbers. In order to conduct the experiments with the typical procedures of LT and HUT, not only the evaluation environments differed, but also the amount of samples presented and the evaluation time interval between samples differed between the two conditions. The present study was approved by the Institutional Review Board of Ewha Womans University (IRB: ewha-202109-0031-04).

### 2.2. Subjects

#### 2.2.1. Almond Beverage Test Subjects

Consumer subjects were recruited through a recruitment flyer on the online bulletin board of the Ewha Womans University website and on-campus flyer distribution. A total of 322 subjects participated in the study, and they comprised young Korean females (mean age: 22.8 ± 3.0 years, 19–38 years) who consumed almond beverages. Each subject selected an experimental group to participate in from the six groups. The numbers of participants in the six groups were as follows: group 1: LT × one-sample test (only target product), n = 53; group 2: LT × two-sample test, n = 54; group 3: LT × four-sample test, n = 54; group 4: HUT × one-sample test, n = 53; group 5: HUT × two-sample test, n = 54; and group 6: HUT × four-sample test, n = 54. Considering that a between-subject design was employed, subjects were allowed to participate in only one experimental group from the six groups. All subjects signed a written consent form prior to the participation.

#### 2.2.2. Vegan Ramen Test Subjects

The recruitment method was similar to that used in the almond beverage test. A total of 287 Korean female subjects who consumed vegan ramen participated in the study. All participants were young Korean female (mean age: 23.3 ± 3.5 years, 19–37 years). Again, participants selected the experimental group to participate in from the six groups. The numbers of participants in the six groups were as follows: group 1: LT × one-sample test (only target product), n = 53; group 2: LT × three-sample test, n = 48; group 3: LT × five-sample test, n = 46; group 4: HUT × one-sample test, n = 50; group 5: HUT × three-sample test, n = 45; and group 6: HUT × five-sample test, n = 45. Subjects were allowed to participate in only one experimental group from the six groups. However, they were allowed to participate in both almond beverage and ramen tests. All subjects signed a written consent form prior to the participation.

### 2.3. Samples and Sample Preparation

#### 2.3.1. Almond Beverage Experiment: Samples and Sample Preparation

Four almond beverage products commercially available in Korea were selected as samples for investigation (Table 1). Among the four products, a target item was selected to directly compare the effects of the evaluation environment and sample number. We selected Almond Breeze Original (ABreeze; Maeil Dairies Co., Ltd., Seoul, Republic of Korea), which received the highest liking score in a preliminary experiment, as the target sample. 

In the LT, all samples were stored in a refrigerator (approximately 5 °C). The samples were subsequently shaken and poured into disposable paper cups (upper diameter: 9 cm, lower diameter: 6 cm, height: 11 cm; OnePojiang Crop., Daegu Metropolitan City, Republic of Korea) and lidded. Approximately 90 ± 5 g of product was served per sample. Subjects received lidded samples and tasted them using straws which is a common way of drinking almond beverage in Korea. A three-digit random number was labeled on each sample cup.

In the HUT, all samples were wrapped in a white sheet to hide the product name and other labeled information and stored at room temperature. Upon receiving the samples, subjects were instructed to store the samples as they normally would for almond beverages (i.e., samples could be stored at room temperature or in a refrigerator). Subjects in groups 5 and 6 who evaluated more than one sample could only taste one sample a day. A time interval ≥1 day between the tasting of different samples was stipulated.

#### 2.3.2. Vegan Ramen Test: Samples and Sample Preparation

The samples used in the ramen consumer test comprised five ramen products commercially available in Korea (Table 2). Similar to the almond beverage experimental protocol, a target sample, namely Delicious Vegan Ramen (SY_M; Samyang Foods, Co., Ltd., Seoul, Republic of Korea), was selected and included in all the conditions. 

In the LT, all samples were stored at room temperature after purchase and prepared according to their respective standard recipes as per manufacturer recommendations. The preparation process, such as the amount of water added, time taken, and order of ingredient addition was strictly controlled. The sample information and preparation methods for LT samples are shown in Table 2. Subjects who participated in the LT received approximately 55 ± 5 g of noodles and 75 ± 5 g of soup for each sample. Each sample was served in a disposable plate (upper diameter: 9.6 cm, lower diameter: 7.8 cm, height: 8 cm; Cleanwrap Corp., Gyeongsangnamdo, Republic of Korea). A three-digit random number was labeled on each sample plate. 

In the HUT, all samples were repackaged using vacuum-packed vinyl and disposable plastic to cover the product name and other labeled information and stored at room temperature. Subjects were allowed to choose their preferred method of sample preparation (i.e., cook their samples as they normally would ramens or according to the standard recipe used in the LT). Unlike the LT subjects, those who participated in the HUT received one intact, repackaged ramen for each sample. A three-digit random number was labeled on each repackaged sample. Additionally, subjects in groups 5 and 6 could only evaluate one sample a day. A time interval ≥1 day between the tasting of different samples was stipulated.

### 2.4. Protocol Design

#### 2.4.1. Laboratory Condition Test (LT)

In the LT, subjects visited the sensory testing laboratory at Ewha Womans University (Seoul, Republic of Korea) for taste testing. They tasted the samples and evaluated their attributes on a paper ballot. 

For the almond beverage test, subjects evaluated each sample based on the following three attributes: overall, taste/flavor, and texture liking using a Korean version of the nine-point hedonic scale [36] (1 = “utterly dislike”, 5 = “neither like nor dislike”, and 9 = “like very much”) translated and validated from the nine-point hedonic scale by Peryam and Pilgrim (1957) [37]. In the one-sample test (group 1), only the target sample was evaluated. In the two-sample test (group 2), sample configuration was obtained by balancing the target sample with one sample from the remaining three samples, and samples were served based on a completely randomized block design. In the four-sample test (group 3), all four samples, including the target sample, were evaluated, and the Williams Latin square method was used to determine the serving order. Spring water was provided to group 2 and 3 subjects who evaluated more than one sample to minimize the physicochemical carryover effect and sensory adaptation. Subjects were allowed 7 min per sample. 

In the ramen test, subjects evaluated each sample based on the following five attributes: overall, appearance, odor/smell, taste/flavor, and texture liking using a Korean version of the nine-point hedonic scale. In the one-sample test (group 1), only the target sample was evaluated. In the three-sample test (group 2), sample configuration was achieved by balancing the target sample with two samples from the remaining four samples, and samples were served based on a completely randomized block design. In the five-sample test (group 3), all five samples, including the target sample, were evaluated, and the Williams Latin square method was used to determine the serving order. Spring water and unsalted crackers (Carr’s Original Table Water, United Biscuits, Carlisle, UK) were provided to group 2 and 3 subjects who evaluated more than one sample to minimize the physicochemical carryover effect and sensory adaptation. Subjects were allowed 10 min per sample. 

#### 2.4.2. Home-Used Test (HUT)

Subjects who participated in the HUT visited the culinary science laboratory at Ewha Womans University to collect the samples to be evaluated at home. The experimenter briefly explained the HUT sample preparation and evaluation procedures to the subjects during sample distribution. Unlike in the LT, subjects were minimally restricted in terms of the taste-testing procedure followed at home, that is, they were allowed to freely choose when to taste and evaluate the samples, what to use for their consumption, how to store them, and whom to eat with. However, as mentioned earlier, HUT subjects were instructed to evaluate only one sample a day, and in so doing, those who received more than one sample were to follow a specific sample evaluation order. 

In the almond beverage HUT, subjects evaluated the same attributes as those evaluated in the LT. Subjects evaluating the target sample only (group 4) had to evaluate the sample within 1 day, those evaluating two samples (group 5) had to evaluate them within 4 days, and those evaluating four samples (group 6) had to evaluate them within 8 days. After completing the questionnaire, subjects either sent it via mail or dropped it in the laboratory ballot box. The sample composition of each experimental group and serving order were similar to those used in the LT. 

Again, the attributes evaluated in the HUT ramen samples were similar to those evaluated in the LT samples. Subjects evaluating the target sample only (group 4) had to evaluate it within 1 day, those evaluating three samples (group 5) had to evaluate them within 6 days, and those evaluating five samples (group 6) had to evaluate them within 10 days. After completing the questionnaire, subjects either sent it via mail or submitted it to the laboratory. The sample composition of each experimental group and serving order was similar to those used in the LT. 

### 2.5. Statistical Analysis

Analysis of variance (ANOVA) using a general linear model (GLM) was performed to investigate the effects of the evaluation environment and sample number on the target sample’s liking scores. Specifically, the following GLM was used: acceptance = evaluation environment + number of evaluation samples + evaluation environment × number of evaluation samples. When the effect was significant, Duncan’s multiple range test was conducted as a post-hoc analysis. Statistical significance was set at α = 0.05. 

For the groups that evaluated multiple samples, the time period between sample tasting differed between the LT and HUT. Thus, the serving order effect on the liking rating was also analyzed. The following model was applied: acceptance = evaluation environment + number of evaluation samples + serving order + evaluation environment × number of evaluation samples + evaluation environment × serving order + number of evaluation samples × serving order + evaluation environment × number of evaluation samples × serving order. 

To analyze the serving order effect, all data were aggregated within each testing environment, except for the one-sample test. We analyzed the serving order effect on the target sample’s liking scores, and the result was a combination of the two- and four-sample tests in the almond beverage experiment and three- and five-sample tests in the ramen experiment. Therefore, the target sample ratings evaluated first, second, third, and fourth in order of tasting differed in the almond beverage test (generally, 1st order ≈ 2nd order > 3rd order ≈ 4th order), and those evaluated first, second, third, fourth, and fifth in order of tasting also varied in the ramen test (generally, 1st order ≈ 2nd order ≈ 3rd order > 4th order ≈ 5th order). Duncan’s multiple range test was performed as a post-hoc analysis when the effect was significant.

Overall, since samples other than the target were evaluated by a relatively small number of panels, statistical analysis was performed exclusively for the target sample. Statistical analyses were performed using IBM SPSS Statistics (version 26; IBM Corp., Chicago, IL, USA) and Microsoft Excel for Microsoft 365 MSO software (Version 2111, Build 16.0.14701.20254; Microsoft, Washington, WA, USA).

## 3. Results

### 3.1. Overall Liking of the Samples

#### 3.1.1. Overall Liking Scores of the Four Almond Beverage Samples

**Figure 2:**
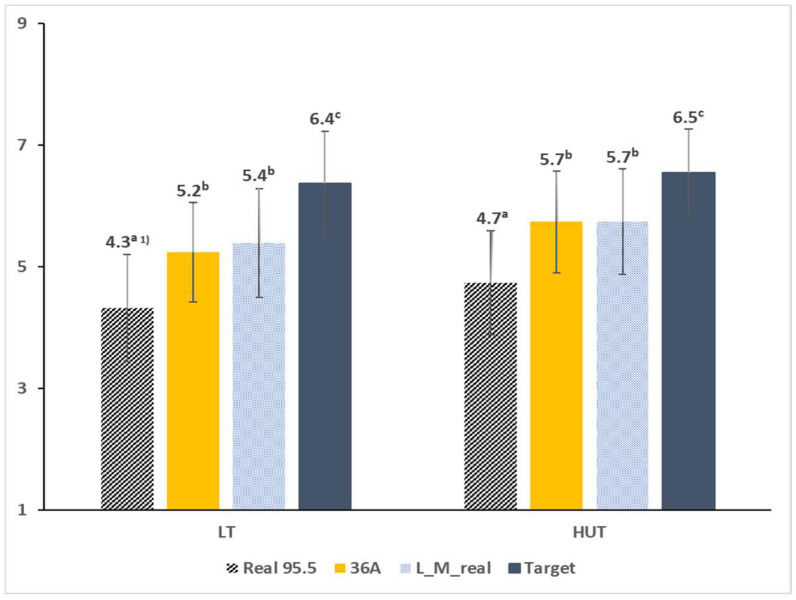
Mean overall liking scores of almond beverage samples in the lab condition test (**left**) and in the home-used test condition (**right**). ^1)^ Within a test environment, different letters indicate significant differences between the mean overall liking values of samples (*p* < 0.001).

ANOVA of the four almond beverage samples revealed significantly different liking scores (*p* = 0.005). Post-hoc analysis of the overall liking scores of the four samples indicated that the target sample received the highest liking score (mean liking scores = target: 6.4, real_95.5: 4.5, 36A: 5.5, and L_M_real: 5.6). When the liking ratings of the four samples were compared separately within each of the two environmental conditions, they differed significantly under both LT and HUT conditions (*p* < 0.001, Figure 2).

#### 3.1.2. Overall Liking Scores of the Five Vegan Ramen Samples

**Figure 3:**
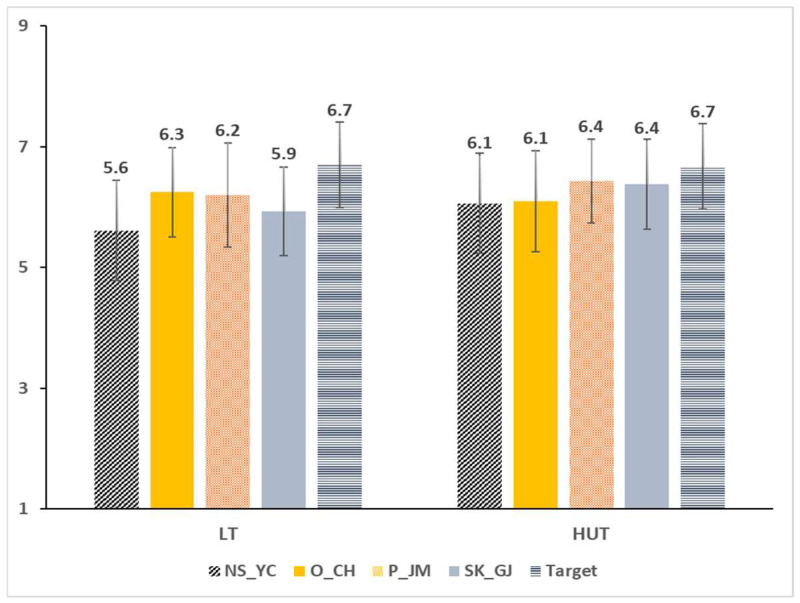
Mean overall liking scores of vegan ramen samples in the lab condition test (**left**) and in the home-used test condition (**right**).

The results of the five ramen samples indicated that their liking scores differed significantly (*p* = 0.007). Post-hoc analysis of the liking scores of the five samples demonstrated that the target sample received the highest liking score (mean liking scores = target: 6.7, NS_YC: 5.8, SK_GJ: 6.1, O_CH: 6.2, and P_JM: 6.3). Sample-type effects were further investigated separately in each of the two environmental conditions. The liking ratings of the five samples did not differ significantly under both LT (*p* = 0.063) and HUT (*p* = 0.142) conditions (Figure 3).

### 3.2. Effect of the Evaluation Environment on the Target Sample’s Liking Scores

#### 3.2.1. The Almond Beverage Target Sample’s Liking Scores by Evaluation Environment

On examining the effect of the evaluation environment, the target sample’s taste/flavor liking was found to be influenced by the evaluation environment (*p* < 0.05, Table 3). However, no significant evaluation environment effect on the overall and texture liking ratings was noted (*p* > 0.05). The HUT taste/flavor score was significantly higher than that of the LT (LT: 6.1; HUT: 6.5).

Additionally, the target sample’s liking ratings were compared between LT and HUT conditions across the groups that evaluated same sample numbers. The target sample’s overall and taste/flavor liking ratings in the one-sample test significantly differed between testing locations. In both cases, HUT scores were significantly higher than LT scores (overall liking scores = LT: 6.1, HUT: 6.5; taste/flavor liking scores = LT: 5.9, HUT: 6.6).

#### 3.2.2. The Vegan Ramen Target Sample’s Liking Scores by Evaluation Environment

The evaluation environment did not significantly influence the liking scores of all target samples (*p* > 0.05, Table 4) when all the data were analyzed together, that is, the target sample’s liking scores were similar regardless of evaluation location. 

The target sample’s liking ratings were compared between the LT and HUT as well as among different sample numbers. Significant differences in the target sample’s appearance liking were noted in the one- and five-sample tests. While the LT appearance liking scores were significantly higher than those of the HUT in the one-sample test (LT: 7.3, HUT: 6.4), they were significantly lower than those of the HUT in the five-sample test (LT: 6.3, HUT: 6.9).

### 3.3. Effect of the Evaluation Sample Number on the Target Sample’s Liking Scores

#### 3.3.1. The Almond Beverage Target Sample’s Liking Scores by Evaluation Sample Number 

In the almond beverage experiment, no significant differences were noted in all attribute liking ratings according to evaluation sample number (*p* > 0.05, Table 3). 

**Figure 4:**
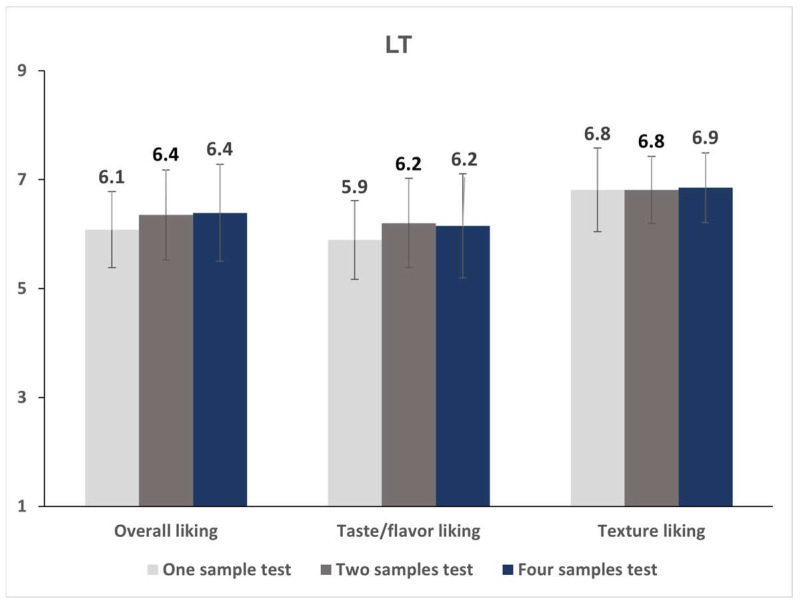
Mean liking scores of the almond beverage test target sample according to the number of evaluation samples in the lab condition test.

**Figure 5:**
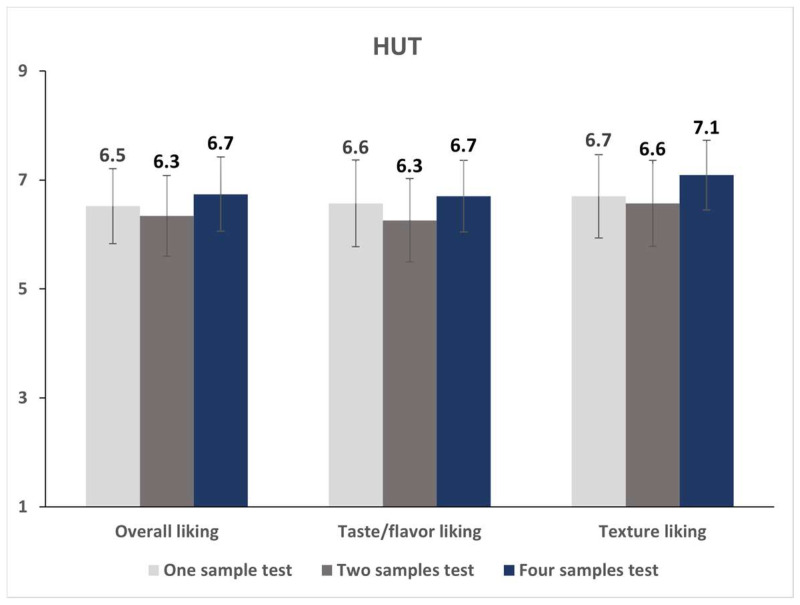
Mean liking scores of the almond beverage test target sample according to the number of evaluation samples in the home-used test condition.

On making separate examinations of the effect of the evaluation sample number by evaluation environment, the sample number had no significant effect on all liking ratings under both LT and HUT conditions (*p* > 0.05, Figure 4 and Figure 5). 

#### 3.3.2. The Vegan Ramen Target Sample’s Liking Scores by Evaluation Sample Number 

Significant differences in the overall, odor/smell, taste/flavor, and texture liking ratings of the target sample were observed when the evaluation sample number was varied (*p* < 0.05, Table 4). The attributes that exhibited a significant sample number effect all yielded significantly higher scores when the target sample was evaluated alone than when it was evaluated with other samples (three- and five-sample tests). The target sample’s liking ratings for different sample numbers were as follows. The overall liking scores were 7.2 (one-sample test) > 6.4 (three-sample test) = 6.4 (five-sample test). The odor/smell liking scores were 7.1 (one-sample test) > 6.2 (five-sample test) ≥ 6.1 (three-sample test). The taste/flavor liking scores were 7.0 (one-sample test) > 6.5 (three-sample test) ≥ 6.4 (five-sample test). Finally, the texture liking scores were 7.4 (one-sample test) > 6.9 five-sample test) ≥ 6.8 (three-sample test).

**Figure 6:**
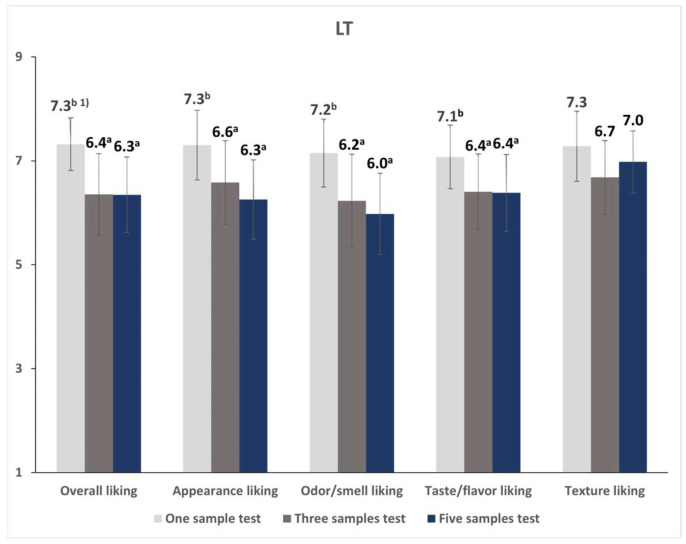
Mean liking scores of the vegan ramen test target sample according to the number of evaluation samples in the lab condition test. ^1)^ Within a sensory attribute, different letters indicate significant differences between the mean values of a different number of evaluation samples (*p* < 0.05).

**Figure 7:**
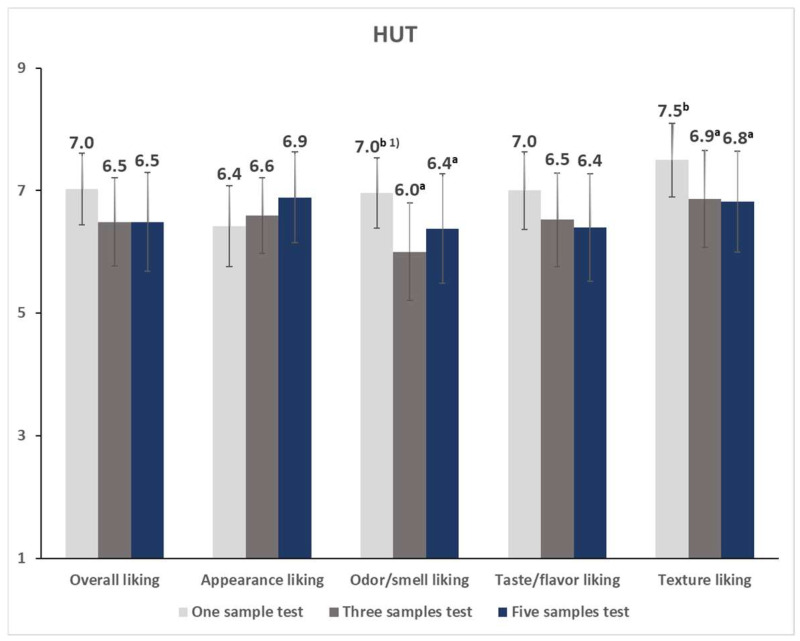
Mean liking scores of the target sample of vegan ramen test according to the number of evaluation samples in the home-used test. ^1)^ Within a sensory attribute, different letters indicate significant differences between the mean values of a different number of evaluation samples (*p* < 0.05).

When the sample number effect was analyzed separately in each evaluation environment, the overall, appearance, odor/smell, and taste/flavor liking scores under LT conditions differed significantly as the number of samples evaluated together was varied (Figure 6). In all cases, the target sample received the highest liking scores in the one-sample test than in other tests. No significant differences were noted between the three- and five-sample tests. Under HUT conditions, the sample number significantly affected odor/smell and texture liking ratings (Figure 7). Consistent with the LT results, the target sample received higher scores when tested alone compared to when tested with other samples. The target sample’s liking scores were similar between the three- and five-sample tests.

Additionally, we analyzed the interaction effect of the environment and sample number, and such an effect was only observed on the appearance liking rating (*p* < 0.05). Although the one-sample test yielded the highest appearance liking score in the LT (Figure 6; one-sample test: 7.3 > three-sample test: 6.6 ≥ five-sample test: 6.3), the five- and one-sample tests produced the highest and lowest scores in the HUT, respectively (Figure 7; five-sample test: 6.9 ≥ three-sample test: 6.6 ≥ one-sample test: 6.4), for the target sample.

### 3.4. Serving Order Effect on the Target Sample’s Liking Scores

#### 3.4.1. Serving Order Effect on the Almond Beverage Target Sample’s Liking Scores

As shown in Table 5, the serving order did not significantly influence the target sample’s liking scores (*p* > 0.05). However, a significant evaluation environment and serving order interaction effect was observed on the overall and taste/flavor liking scores (*p* < 0.05). For further elucidation, the serving order effect was analyzed separately in each evaluation environment. The results indicated that the overall and taste/flavor scores of the LT were significantly influenced by the serving order, and the highest scores were obtained when the target sample was tasted last (*p* < 0.05, overall liking: 5.8–7.3 points; taste/flavor liking: 5.6–7.1 points). However, all HUT liking ratings were not significantly influenced by the serving order (*p* > 0.05).

#### 3.4.2. Serving Order Effect on the Vegan Ramen Target Sample Liking Scores

Although the results revealed no significant differences in all target sample liking scores according to serving order (Table 6, *p* > 0.05), odor/smell likings were affected by the interaction between the evaluation environment and serving order (*p* < 0.05). Moreover, the results indicated a marginal environment × serving order effect on the overall (*p* = 0.071) and appearance (*p* = 0.082) liking ratings. Thereafter, we categorized the liking scores according to the evaluation environment and analyzed the serving order effect. The LT results initially revealed significant differences in the overall, appearance, odor/smell, and taste/flavor liking scores by serving order (*p* < 0.001). All the attributes yielded their highest and lowest scores when the target sample was evaluated first and fifth (last), respectively (overall liking: first: 7.3, fifth: 5.4; appearance liking: first: 7.2, fifth: 5.9; odor/smell liking: first: 7.3, fifth: 5.2; and taste/flavor liking: first: 7.1, fifth: 5.6). However, the target sample’s serving order did not significantly affect the HUT results (*p* > 0.05).

## 4. Discussion

The present study investigated the effects of the evaluation environment and number of samples evaluated within a session on the target sample’s liking scores. The study comprised two independent consumer taste experiments: the almond beverage and vegan ramen evaluation tests. In both experiments, sample liking ratings were evaluated under either LT or HUT conditions. Additionally, the sample number varied (almond beverage: one, two, and four samples; ramen: one, three, and five samples) in different testing groups. The results demonstrated that target sample ratings were influenced more by the number of samples evaluated within a session than the tasting environment. The effect of the evaluation sample number yielded different results depending on the product type. In the almond beverage test, no significant changes were noted in the target sample liking ratings according to sample number; nonetheless, significant differences were observed in the liking ratings of the target ramen sample. 

### 4.1. Effect of the Evaluation Environment on the Target Sample’s Liking Scores

The results revealed that the evaluation environment did not strongly influence the target sample’s liking ratings in both the almond beverage and ramen tests. In the almond beverage test, only the taste/flavor liking of the target sample significantly differed between the LT and HUT (LT: 6.1; HUT: 6.5), and no evaluation environment effect was observed on all liking ratings of the target ramen sample. Several studies have investigated the effect of the evaluation environment on liking ratings, and different results have been obtained depending on the product item. While studies have shown a significant evaluation environment effect on the liking ratings for crackers, milk [22], chocolate bars [16], yogurt [21], and high-fat cream cheese [10], no significant evaluation environment effect has been observed on pizza [7], sparkling water [22], and low-fat cream cheese [10]. In our study, the evaluation environment had negligible influence on the liking ratings of both almond beverage and ramen samples.

When the effect of the evaluation environment on the target sample’s liking ratings for the same sample number was analyzed, differences were noted in the appearance and taste/flavor liking ratings of the one-sample almond beverage test and appearance liking ratings of the one- and five-sample ramen tests. Overall, among the four attributes that were significantly affected by the evaluation environment, three were observed in the one-sample test. A previous study reported that hedonic sample differentiation was less pronounced when samples were evaluated first in order of tasting [38] because consumers were not accustomed to using the scale [39], and potentially used the sample evaluated first to familiarize themselves with the scale [40]. Since the one-sample test was relatively affected by the evaluation environment than other sample number conditions, this result indicates that the sample evaluated first in order of tasting exhibits relatively different hedonic results.

### 4.2. Effect of the Sample Number on the Target Sample’s Liking Scores

The effect of sample number on the target sample liking ratings within a tasting session was food-product-dependent, that is, sample number did not significantly affect almond beverage liking ratings; however, it did strongly influence ramen liking ratings. These contrasting outcomes between the two food items may be due to the different levels of flavor complexity. Porcherot and Issanchou (1998) found sample liking ratings to potentially change under different conditions, more so if the sample possessed higher flavor complexity [32]. The higher flavor complexity of ramen than that of almond beverage might have induced the fluctuation of ramen liking ratings as a function of sample number within a tasting session. Regarding the ramen test results, a significant sample number effect was observed on the overall, odor/smell, taste/flavor, and texture liking ratings. On assessing all attributes that were subjected to the sample number effect, the target sample received significantly higher scores when evaluated individually than when evaluated with other samples (overall target sample liking scores: 7.2 (one-sample test) > 6.4 (three-sample test) = 6.4 (five-sample test); odor/smell liking scores: 7.1 (one-sample test) > 6.2 (five-sample test) ≥ 6.1 (three-sample test); taste/flavor liking scores: 7.0 (one-sample test) > 6.5 (three-sample test) ≥ 6.4 (five-sample test); texture liking scores: 7.4 (one-sample test) > 6.9 (five-sample test) ≥ 6.8 (three-sample test)). The first-position effect, that is, the tendency of a sample evaluated first in order of tasting to receive higher scores than those evaluated at latter positions, has been explained in several previous studies [27,41,42]. The first sample often receives the highest score because consumers tend to be subjected to fatigue when evaluating multiple samples [2]; moreover, the contrast effect, which manifests when evaluating a sample together with other sample types, can also result in decreased liking ratings. These findings suggest that high-complexity foods are susceptible to sample number influence, thus again corroborating the fact that the one-sample test potentially yields different results from multiple-sample tests.

On analyzing the sample number effect separately according to the evaluation environment, the almond beverage target sample liking ratings were not influenced by sample number in both environmental conditions, but ramen target sample liking ratings were affected by sample number in both testing locations. When analyzing the hedonic ratings according to evaluation environment, the hedonic ratings of the other three almond beverage samples were significantly different from that of the target in both LT and HUT, but hedonic levels of other four ramen samples were similar with that of the target in both environments. Kamenetzy (1959) observed that the hedonic ratings of the sample with high preference remained regardless of the conditions and the samples evaluated together [28]. The target of the almond beverage experiment which was the significantly preferred sample over the other samples showed consistent ratings regardless of different conditions of sample number in both LT and HUT. On the contrary, the ramen target sample which showed a relatively similar liking level with other samples received significantly different ratings in different sample number conditions. Furthermore, ramen target sample liking ratings were strongly affected by sample number under LT conditions (four significant attributes among five) and relatively less affected under HUT conditions (two significant attributes among five). This ramen sample difference observed between the HUT and LT may be attributed to differences in the sample evaluation procedures followed in the two tests. Subjects who participated in the LT were instructed to taste three or five samples sequentially in one session, whereas HUT subjects were asked to prepare and taste one sample a day. The short interval between the tasting of different samples in the LT might have induced strong “carry over” and contrast effects, since a more direct comparison could be made between samples in the LT than in the HUT [43]. The presence of a strong serving order effect in ramen testing but not in almond beverage testing may also be due to the sensory-specific satiety. Sensory-specific satiety refers to the hedonic decline in similar flavor quality upon repeated consumption [44]. The studies have reported that SSS may be present in higher level when the sample of interest carries stronger flavor or texture quality [45,46]. A previous study found that noise-induced rating variability increased more with the sequential monadic design than with the pure monadic design as the evaluated sample number per session increased [5].

### 4.3. Effect of the Serving Order on the Target Sample’s Liking Scores

Based on the finding wherein a stronger carry over/contrast effect potentially existed in the LT than in the HUT, the serving order effect on the target sample’s liking ratings was also analyzed, with results revealing a significant serving order effect in the LT, but not in the HUT, in both the almond beverage and ramen tests. The presence of a serving order effect exclusively under LT conditions further supports the fact that the time interval between different sample evaluations is critical to maximize or minimize the carry over effect during sample evaluation because the contrast effect and sensory fatiguing may occur easily when multiple samples are evaluated within a single session. Previous studies have reported that when using the pure monadic design, subjects can evaluate samples based on personal preference because their perception of one sample is not easily affected by that of other samples [22], and similarly, analyzing a single product in one session would be more appropriate when considering the contextual effect [41]. When using the pure monadic design, which is frequently utilized in the HUT, the target sample ratings exhibit more consistent rating patterns regardless of the number and types of other samples that are also evaluated.

The present study has several limitations to its generalizability. First, subjects were restricted to Korean women. We considered female subjects to be more appropriate for this study because women generally tend to consume vegan foods more than men [47]. However, due to this restriction, the generalization and interpretation of this study’s findings is limited. In addition, each experimental group had a relatively small number of subjects. If more consumers, including male subjects, were to be recruited, the results would be more convincing.

## 5. Conclusions

The present study investigated the effect of the evaluation environment on the liking ratings for different sample numbers. Almond beverage and vegan ramen were chosen for this investigation, and in each experiment, a target sample was specifically selected to investigate the effects of the evaluation environment and sample number. Our key findings were as follows: 1. The evaluation environment’s effect on liking ratings was not significantly influential in both the almond beverage and ramen tests. 2. The sample number effect on liking ratings was food-product-dependent (no sample number effect was observed in the almond beverage test, but the effect was strong in the ramen test), and the effect was more prominent in the LT than in the HUT. 3. The serving order effect was considerably present in the LT, but not in the HUT, in both the almond beverage and ramen experiments. Based on this study’s results, the testing environment condition and number of samples evaluated together should be carefully chosen depending on the properties of the product of interest. The HUT is potentially more efficacious than the LT, as the latter was more easily affected by sequential bias and the concurrent evaluation of multiple samples, especially in terms of products with complex flavors.

## Figures and Tables

**Table 1:** Almond beverage samples information.

Sample Code	Product Name	City/Nation	Manufacturer
Target (Abreeze)	Almond breeze original	Gwanju, Republic of Korea	Mail Dairies Co., Ltd.
Real 95.5	Real almond 95.5	Pyeongtaek, Gyeonggi-do, Republic of Korea	Hanmi Healthcare Inc.
36A	36 almond original	Cheonan, Chungcheongnam-do, Republic of Korea	Sahmyook Foods Co., Ltd.
L_M_real	Light minute real almond	Damyang, Jeollanam-do, Republic of Korea	Nature & People Co., Ltd.

**Table 2:** Vegan ramen samples information and standard recipe.

Sample Code	Product Name	City/Nation	Manufacturer	Standard Recipe of Ramens Used in LT
Target (SY_M)	Samyang delicious vegan ramen	Seoul, Republic of Korea	Samyang Foods Co., Ltd.	1. Boil 550 g of water. 2. When the water boils, add the soup powder and noodles, flakes, and seasoning oil together. 3. Boil for 5 min.
NS_YC	Nongshim vegetable ramen	Seoul, Republic of Korea	Nongshim Co., Ltd.	1. Boil 500 g of water. 2. When the water boils, add the soup powder, noodles, and flakes together. 3. Boil for 4 min and 30 s.
O_CH	Ottogi veggie noodle soup chaehwang	Pyeongtaek, Gyeonggi-do, Republic of Korea	Ottogi Co., Ltd.	1. Add the flakes to 500 g of water and boil the water. 2. When the water boils, add the soup powder and noodles. 3. Boil for 3 min.
P_JM	Pulmuone right noodle	Eumseong, Chungcheongbuk-do, Republic of Korea	Pulmuone Co., Ltd.	1. Boil 500 g of water. 2. When the water boils, add the soup powder and noodles, flakes, and seasoning oil together. 3. Boil for 4 min and 30 s.
SK_GJ	Sahmyook vegetable potato noodle	Wanju, Jeollabuk-do, Republic of Korea	Saerom Food., Co., Ltd.	1. Boil 550 g of water. 2. When the water boils, add the soup powder, noodles, and flakes together. 3. Boil for 4 min and 30 s.

**Table 3:** Effect of the evaluation environment and the number of evaluation samples on the liking ratings for the almond beverage test target sample.

Factor	Attributes	F-Value	*p*-Value
Evaluation environment (A)	Overall liking	3.052	0.082
	Taste/flavor liking	5.945	0.015
	Texture liking	0.066	0.797
Number of evaluation samples (B)	Overall liking	0.738	0.479
	Taste/flavor liking	0.556	0.574
	Texture liking	1.181	0.308
A × B	Overall liking	0.935	0.394
	Taste/flavor liking	1.169	0.312
	Texture liking	0.865	0.422

**Table 4:** Effect of the evaluation environment and the number of evaluation samples on the liking ratings for the vegan ramen test target sample.

Factor	Attributes	F-Value	*p*-Value
Evaluation environment (A)	Overall liking	0.003	0.959
	Appearance liking	0.227	0.634
	Odor/smell liking	0.001	0.969
	Taste/flavor liking	0.014	0.907
	Texture liking	0.239	0.625
Number of evaluation samples (B)	Overall liking	9.769	<0.001
	Appearance liking	1.292	0.276
	Odor/smell liking	11.527	<0.001
	Taste/flavor liking	5.880	0.003
	Texture liking	5.368	0.005
A × B	Overall liking	0.834	0.435
	Appearance liking	7.002	0.001
	Odor/smell liking	1.220	0.297
	Taste/flavor liking	0.108	0.898
	Texture liking	0.512	0.600

**Table 5:** Effect of the evaluation environment, the number of evaluation samples and the serving order on the liking ratings for the two-sample and four-sample conditions of the almond beverage test target samples.

Factor	Attributes	F-Value	*p*-Value
Evaluation environment (A)	Overall liking	0.071	0.791
	Taste/flavor liking	0.949	0.331
	Texture liking	0.012	0.912
Number of evaluation samples (B)	Overall liking	0.069	0.793
	Taste/flavor liking	0.279	0.598
	Texture liking	0.186	0.667
Serving order (C)	Overall liking	1.626	0.184
	Taste/flavor liking	2.211	0.088
	Texture liking	0.884	0.450
A × B	Overall liking	3.591	0.060
	Taste/flavor liking	3.988	0.047
	Texture liking	2.316	0.130
A × C	Overall liking	4.575	0.004
	Taste/flavor liking	3.656	0.013
	Texture liking	1.779	0.152
B × C	Overall liking	0.703	0.403
	Taste/flavor liking	0.529	0.468
	Texture liking	0.050	0.823
A × B × C	Overall liking	0.435	0.510
	Taste/flavor liking	0.961	0.328
	Texture liking	1.862	0.174

**Table 6:** Effect of the evaluation environment, the number of evaluation samples, and the serving order on the liking ratings for the three-sample and five-sample conditions of the vegan ramen test target samples.

Factor	Attributes	F-Value	*p*-Value
Evaluation environment (A)	Overall liking	1.519	0.220
	Appearance liking	3.417	0.066
	Odor/smell liking	1.053	0.306
	Taste/flavor liking	0.442	0.507
	Texture liking	0.075	0.784
Number of evaluation samples (B)	Overall liking	0.011	0.918
	Appearance liking	0.043	0.836
	Odor/smell liking	0.333	0.565
	Taste/flavor liking	0.169	0.682
	Texture liking	1.176	0.280
Serving order (C)	Overall liking	1.599	0.177
	Appearance liking	0.342	0.850
	Odor/smell liking	0.619	0.649
	Taste/flavor liking	1.123	0.348
	Texture liking	0.939	0.443
A × B	Overall liking	0.938	0.334
	Appearance liking	0.364	0.547
	Odor/smell liking	0.206	0.650
	Taste/flavor liking	0.789	0.376
	Texture liking	1.799	0.182
A × C	Overall liking	2.198	0.071
	Appearance liking	2.104	0.082
	Odor/smell liking	4.070	0.004
	Taste/flavor liking	1.649	0.164
	Texture liking	0.381	0.822
B × C	Overall liking	2.934	0.056
	Appearance liking	2.921	0.057
	Odor/smell liking	0.990	0.374
	Taste/flavor liking	3.219	0.042
	Texture liking	1.158	0.317
A × B × C	Overall liking	0.615	0.542
	Appearance liking	0.214	0.808
	Odor/smell liking	0.392	0.677
	Taste/flavor liking	0.434	0.649
	Texture liking	0.783	0.459

## Data Availability

Data can be made available upon request.
